# Phosphorylated-p38 mitogen-activated protein kinase expression is associated with clinical factors in invasive breast cancer

**DOI:** 10.1186/s40064-016-2636-0

**Published:** 2016-06-30

**Authors:** Bin Wang, Huayong Jiang, Ning Ma, Yajie Wang

**Affiliations:** Department of Oncology, Changhai Hospital, Second Military Medical University, 168, Changhai Road, Shanghai, 200433 China; Department of Radiation Oncology, Beijing Military General Hospital, Beijing, 100700 China; Clinical Laboratory, 85th Hospital of PLA, Shanghai, 200052 China

**Keywords:** Phosphorylate, p38 MAPK, Invasive, Breast cancer

## Abstract

**Purpose:**

P38 mitogen-activated protein kinases (MAPK) level is an important prognostic factor in breast cancer. This study was performed to detect the expressions of P-p38 MAPK expression in breast cancer and explore their correlations with clinicopathological factors.

**Experimental design:**

Tumor samples from 355 Chinese patients diagnosed with invasive breast cancer and adjacent non-cancerous tissue were collected between 2003 and 2010. The expression of P-p38 MAPK was analyzed using immunohistochemical staining. The correlations between P-p38 MAPK expression and clinicopathological findings including age, AJCC Stage, Histologic characters, ER, PR, and HER2 were analyzed using the parametric correlation method. P-p38 MAPK was selected as dependent variable to perform multivariate analysis respectively at last.

**Results:**

Overall, 161 (45 %) and 183 (52 %) of the 355 specimens showed positive P-p38 MAPK staining in the cytoplasm and nucleus respectively, which were significant higher than that in the adjacent non-cancerous tissues in both the cytoplasm and the nucleus. High P-p38 MAPK expression of cytoplasm and nucleus were both associated with positive PR status in luminal A/B type of breast cancer, and were both associated with positive HER2 status in HER2-positive type of breast cancer. The result of multivariate analysis demonstrated that HER2 and PR were both significantly association with P-p38 MAPK expression of cytoplasm and nucleus.

**Conclusions:**

Our study suggests that P-p38 MAPK expression were significantly associated with clinicopathological factors and PR/HER2 might association with phosphorylation of p38 MAPK in different types of breast cancer.

## Background

According to the May Clinic, breast cancer is the second most common cancer after skin cancer in women in the United States and the most frequently diagnosed malignancy among women worldwide (incidence, approximately 1.38 million per year) that results in 46 million deaths (Lee et al. 2014; Jemal et al. 2011). Activation of mitogen-activated protein kinases (MAPK) is one of the most important intracellular signal transduction pathways in breast carcinoma progression (Blenis 1993; Crews and Erikson 1993).

The MAPK family includes extracellular signal-regulated kinase (ERK), c-Jun N-terminal kinase (JNK), and P38 MAPK (Lei et al. 2014). As a member of the MAPK family, P38 MAPK has been identified as the critical point for a normal immune and inflammatory response as the point of convergence of intracellular downstream growth factor receptor signaling (Cuenda and Rousseau 2007) and the level of them is reportedly associated with advanced stage and short survival in cancers of the breast (Esteva et al. 2004), prostate (Park et al. 2003; Khandrika et al. 2009), bladder (Kumar et al. 2010), liver (Iyoda et al. 2003), and lung (Greenberg et al. 2002; Koul et al. 2013). Activation of P38 MAPK could result in induction of gene expression, leading to increased proliferation, invasion, and metastasis in solid cancer (Koul et al. 2013; Mo et al. 2012) and endogenous p38 MAPK activity also reportedly correlates well with breast carcinoma cell invasiveness (Huang et al. 2000; Tang and Han 2013).

The activated form of p38 MAPK, phosphorylation of p38 MAPK (P-p38 MAPK) has been reported in response to ultraviolet irradiation, biologic inducers (e.g., growth factors and cytokines), and chemicals (Dérijard et al. 1994). Levels of P-p38 MAPK have been reported to elevate in breast cancer cells and may be associated with poor overall survival (OS) in patients with lymph node–positive breast carcinoma with adjuvant chemotherapy while location of P-p38 MAPK was not mentioned (Esteva et al. 2001, 2004). Davidsone et al. (2006) also showed that a higher p38 activation ratio correlated with shorter OS in a total of 19 cases. Even so, the expression of P-p38 MAPK in breast cancer and the associated with clinical factors were still not well studied due to a lack of sufficient data.

Based on a total of 355 patients with primary invasive breast carcinoma, the primary goal of the current study was to explore the prevalence of P-p38 MAPK expression and their possible roles in breast cancer. Our observation might direct further investigation on the functions of P-p38 MAPK and influence factor of its expression so as to guide the treatment of invasive breast cancers in future.

## Methods

### Ethics statement

All of the specimens used in this study were used after each patient provided written informed consent. The Ethics Committee of Changhai Hospital granted study approval. The authors confirm that necessary consent was obtained from every patient involved in this study for both participation and publication.

### Study population

Specimens of patients who were diagnosed with primary invasive breast cancer and underwent a surgical procedure between January 2003 and December 2010 at the First Affiliated Hospital of Second Military Medical University (Changhai Hospital, Shanghai, China) were evaluated. Each tumor was classified according to the tumor-node-metastasis (TNM) classification [American Joint Committee on Cancer (AJCC) stage]. Patient age at diagnosis, menopausal status, largest tumor diameter, number of lymph node metastases, TNM stage (AJCC), histological type, and histology grade (Elston-Ellis grade) were recorded with ER, PR, and HER2 expressions. All of the included patients were treated according to National Comprehensive Cancer Network guidelines, which recommend the use of anthracycline-based regimens or anthracycline + taxane-based regimens according to lymph node metastasis status. Anti-hormone and anti-HER2 therapy for patients was used according to ER and HER2 expression status.

Among those cases, patients with stage IV disease, a non-curative resection, or another primary tumor site and those who received preoperative radiotherapy or chemotherapy were excluded. Patients who were not treated according to these guidelines were also excluded. We ultimately included a total of 300 patients with invasive breast cancer in this study. The median patient age was 53 (range 31–84) years, while the median OS was 56 (range 3–115) months.

### Tissue microarray and immunohistochemistry

Paraffin-embedded pathological specimens were obtained from the surgically resected tissues. All of these resection samples were treated with a standard fixation, dissection, and processing protocol. In addition, 300 pericarcinoma tissue samples were collected as a control group.

To examine the typical pathological changes, a large tissue microarray (TMA) was used. TMA blocks were constructed using a tissue arraying instrument (Beecher Instruments, Sun Prairie, WI, USA). Cylinders (1.5 mm in diameter) of representative areas of a tissue block were punched from the center of the tumor away from the areas of ulceration and necrosis and re-embedded in a defined position within a recipient paraffin block. The TMA blocks were then cut into 4-mm sections and processed for immunohistochemistry (IHC).

After being washed with phosphate-buffered saline, the specimens were incubated with the primary antibody using ER antibody (dilution 1:50), PR antibody (dilution 1:50), HER2 antibody (dilution 1:50), and P-p38 MAPK antibody (dilution 1:40; Cell Signaling Technology Inc. Boston, USA). Immunostaining was conducted using the Envision System with diaminobenzidine (Dako, Glostrup, Denmark). A negative control was obtained by replacing the primary antibody with normal murine or rabbit immunoglobulin G with the same dilution.

### IHC evaluation

The P-p38 MAPK expressions in the TMA were independently evaluated by three individuals (Bin Wang, Huayong Jiang, and Ning Ma) who were blinded to the patients’ clinicopathological data. Discrepancies were resolved by consensus among the three evaluators.

A positive HER2 result was determined by IHC staining of 3+ or of 2+ with a positive fluorescent in situ hybridization result. P-p38 MAPK, ER, and PR receptors were all graded by the three independent observers using a four-point scale on which 0 = no staining, 1+ = light staining, 2+ = moderate staining, and 3+ = strong staining (Leake et al. 2000; Mohammed et al. 2012). Slices with a score of 1+ were classified as having “low expression,” those with 2+ as “high expression,” and those with 3+ as “overexpression” (“positive”) in contrast to slices with a score of 0, which were classified as having “no expression” (“negative”).

### Statistical analysis

The correlation analysis was used to find the possible association between P-p38 MAPK expression and the clinicopathological parameters. The Mann–Whitney U test was used to analyze the correlation between P-p38 MAPK expression and patient age. Kaplan–Meier curves were plotted to assess the effect of P-p38 MAPK expression on OS. Different survival curves were compared using the log-rank test. Multivariate proportional Cox models were used to assess the prognostic significance of P-p38 MAPK expression, age, tumor stage, histological type, and HER2, ER, and PR expressions. *P* values ≤0.05 were considered significant. The statistical analysis was performed using SPSS19.0 software (SPSS, Inc.).

## Results

### Staining of P-p38 MAPK in breast cancers

**Fig. 1:**
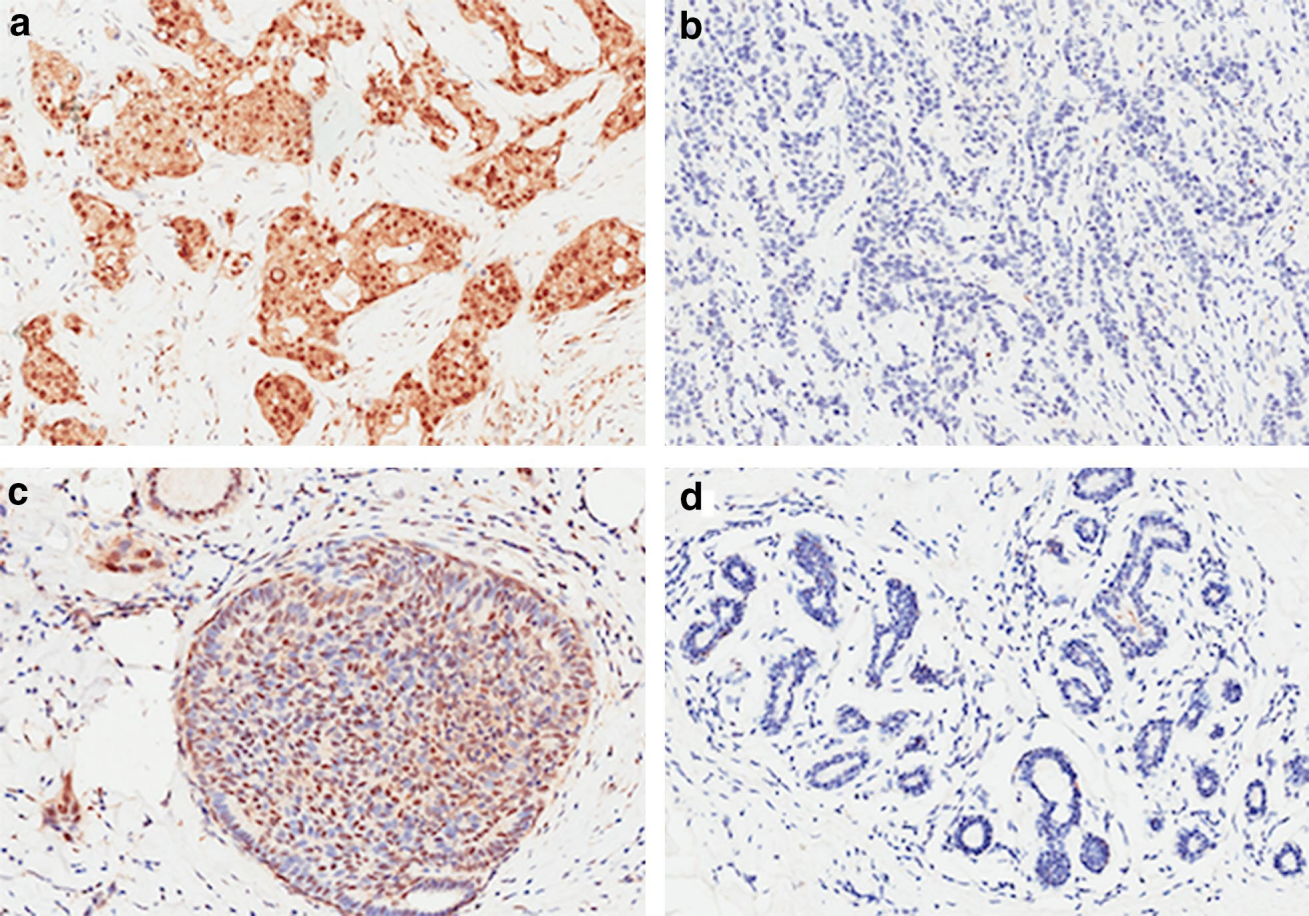
Representative of breast cancer immunostaining for the P-p38 MAPK in the tumor tissues (**a**, **b**) and adjacent non-cancerous tissues (**c**, **d**). **a** Positive for breast cancer tissues. **b** Negative for breast cancer tissues. **c** Positive for adjacent non-cancerous tissues. **d** Negative for adjacent non-cancerous tissues

P-p38 MAPK was expressed in the cytoplasm and nucleus but not in the membrane (Fig. 1). The expression level of P-p38 MAPK in the cytoplasm and nucleus were both calculated. Of all 355 cases, positive P-p38 MAPK staining was seen in the cytoplasm of 161 (45 %) and in the nucleus of 183 (52 %) (*P* = 0.421 between them). P-p38 MAPK expression levels in the cytoplasm and nucleus were both compared between in tumor tissues and adjacent non-cancerous tissues. Positive P-p38 MAPK staining was found in the cytoplasm in 115 (32 %) and in the nucleus in 133 (37 %) of all examined pericarcinoma tissue samples. P-p38 MAPK expression was higher in the tumor tissues than that in the adjacent non-cancerous tissues in both the cytoplasm and the nucleus (*P* < 0.001 and <0.001, respectively; Table 1).

**Table 1:** Diffrence of P-p38 MAPK expression between cancerous tissues and non-cancerous tissues

	N	Negative (%)	Positive (%)	*P*
P-p38 MAPK of cytoplasm				
Cancerous tissues	355	194 (55)	161 (45)	<0.001
Pericarcinoma tissues	355	240 (68)	115 (32)	
P-p38 MAPK of nucleus				
Cancerous tissues	355	172 (48)	183 (52)	<0.001
Pericarcinoma tissues	355	222 (63)	133 (37)	

### Association between P-p38 MAPK expression and clinicopathological parameters

The correlation between P-p38 MAPK expression and clinicopathological parameters was also examined to further elucidate its prognostic value in invasive breast cancer. We found a significant correlation between P-p38 MAPK expression and lymph node metastasis status and AJCC stage in the cytoplasm and nucleus (*P* = 0.012 and 0.028 for lymph node metastasis status, and *P* = 0.032 and 0.047 for AJCC stage respectively) but not between P-p38 MAPK expression and age, menopause status, depth of invasion, histological classification and histological type (*P* > 0.05; Table 2).

**Table 2:** Correlations between P-p38 MAPK expression and clinicopathologic parameters

Factors	P-p38 MAPK of cytoplasm									
	Negative	Positive	*R*	*P*	Luminal A/B (n = 197)		HER2-positive (n = 74)		Triple negative (n = 84)	
					*R*	*P*	*R*	*P*	*R*	*P*
Median age (years)										
Median (range)	53 (30–81)	53 (31–81)	0.037	0.357	0.001	0.988	0.017	0.848	0.176	0.036
Menopause										
Yes	103	90	0.002	0.687	−0.039	0.558	−0.018	0.87	0.19	0.058
No	91	71								
Depth of invasion										
T1	73	57	0	0.999	0.042	0.517	−0.063	0.543	−0.03	0.759
T2/T3/T4	121	104								
Regional lymph node metastasis										
Negative (N0)	60	93	0.016	0.72	0.027	0.662	0.026	0.795	−0.024	0.807
Positive (N1/N2/N3)	134	68								
Stage										
I	60	49	−0.077	0.098	−0.046	0.463	−0.158	0.127	−0.088	0.365
II/III	134	112								
Histologic grade										
I	13	10	−0.034	0.474	0.003	0.961	−0.204	0.057	−0.067	0.5
II/III	181	151								
Histologic type										
Ductal	182	148	0.009	0.85	−0.041	0.533	0.147	0.173	−0.011	0.912
Lobular	12	13								
ER										
Negative	86	77	0.013	0.775	0.148	0.025	–	–	–	–
Positive	108	84								
PR										
Negative	122	93	0.083	0.078	0.22	0.001	–	–	–	–
Positive	72	68								
Her2										
Negative	108	93	0.015	0.745	0.029	0.646	0.31	0.003	–	–
Positive	86	68								
P-p38 MAPK of nucleus										
Negative	163	9	0.772	0.000	0.784	0.000	0.812	0.000	0.717	0.000
Positive	31	152								

Factors	P-p38 MAPK of nucleus									
	Negative	Positive	*R*	*P*	Luminal A/B (n = 197)		HER2-positive (n = 74)		Triple negative (n = 84)	
					*R*	*P*	*R*	*P*	*R*	*P*
Median age (years)										
Median (range)	53 (30–59)	53 (31–81)	0.028	0.479	0.012	0.817	0.037	0.684	0.099	0.234
Menopause										
Yes	95	98	−0.019	0.691	−0.043	0.507	0.005	0.965	0.042	0.671
No	77	85								
Depth of invasion										
T1	60	70	−0.026	0.586	0.005	0.935	−0.042	0.684	−0.059	0.542
T2/T3/T4	112	113								
Regional lymph node metastasis										
Negative (N0)	74	79	−0.019	0.68	−0.033	0.584	0.045	0.653	−0.031	0.747
Positive (N1/N2/N3)	98	104								
Stage										
I	49	60	−0.087	0.061	−0.068	0.272	−0.108	0.295	−0.129	0.182
II/III	123	123								
Histologic grade										
I	10	13	−0.092	0.05	−0.052	0.408	−0.135	0.21	−0.143	0.148
II/III	162	170								
Histologic type										
Ductal	160	170	−0.005	0.924	−0.063	0.333	0.212	0.05	−0.073	0.462
Lobular	12	13								
ER										
Negative	82	81	0.07	0.127	0.122	0.053	–	–	–	–
Positive	90	102								
PR										
Negative	116	99	0.171	0.000	0.225	0.000	–	–	–	–
Positive	56	84								
Her2										
Negative			−0.006	0.894	−0.018	0.771	0.257	0.013	–	–
Positive	82	72								
P-p38 MAPK of nucleus										
Negative			–	–	0.784	0.000	0.812	0.003	0.727	0.000
Positive	–	–								

### Association between P-p38 MAPK expression and ER, PR, or HER2 expression

ER, PR and HER2 are the four well-known critical factors in breast cancer that guide its clinical treatment and prognosis. What is interesting that upon analyzing the association between P-p38 MAPK and ER, PR and HER2 expression in overall breast cancer, we found the positive correlation between P-p38 MAPK in nucleus and PR (*P* = 0.000, R = 0.171), while did not found any positive correlation thing between P-p38 MAPK and ER, PR or HER2 in cytoplasm (Table 2).

These three parameters mentioned above are also most important in determining the four types of breast cancer types including ER-positive [luminal A subtype, luminal B subtype], HER2-positive subtype, and triple-negative subtype. Then the subtypes of breast cancer were studied one by one. We know that PRs are the products of the connection between estrogen and its receptor. As such, ER-positive breast cancer (luminal A and luminal B) was the focus of our observation. We then attempted to determine the correlation between P-p38 MAPK and PR expression in these two subtypes of breast cancer. Interestingly, we found that this kind of positive correlation did exist both in cytoplasm and in the nucleus (*P* = 0.001, R = 0.220 in the cytoplasm and *P* = 0.000, R = 0.225 in the nucleus) while with ER (*P* = 0.025, R = 0.148) only in the cytoplasm.

What`s more, we also found a positive correlation between P-p38 MAPK expression and HER2 (*P* = 0.003, R = 0.310 in the cytoplasm and *P* = 0.013, R = 0.257 in the nucleus) in HER2-positive subtype. Nothing significant correlation was found in triple-negative breast cancer.

ER+HER2+ luminal B breast cancer was then studied to clarify the correlation between P-p38 MAPK and PR or HER2 at last. As a result, we did find a positive correlation between P-p38 MAPK expression and PR (*P* = 0.002, R = 0.294 in the cytoplasm and *P* = 0.001, R = 0.302 in the nucleus) while not HER2 (*P* = 0.062, R = 0.185 in the cytoplasm and *P* = 0.219, R = 0.121 in the nucleus).

### Multivariate logistic regression analysis

Because TNM stage was dependent on tumor size and axillary lymph node metastasis, it was excluded in multivariate analysis. Variables that were selected for multivariate analysis included P-p38 MAPK in cytoplasm, P-p38 MAPK in nucleus, HER2, ER, PR, age at diagnosis, menopause, histology grade, histology type, depth of invasion (T), and regional lymph nodes (N). As Table 3 showed, Only PR was demonstrated a significant association with P-p38 MAPK in cytoplasm and P-p38 MAPK in nucleus in overall breast cancer and PR was also demonstrated the only one which was a significant association in luminal A/B breast cancer. As a result, HER2 was also demonstrated a significant correlated with P-p38 MAPK in HER2-Positive type breast cancer.

**Table 3:** Multivariate analysis by logistic regression analysis

Breast cancer	Dependent variable	Independent variable	SE	P value	OR	95 % CI
Overall	P-p38 MAPK of cytoplasm	PR	0.234	0.008	1.866	1.178–2.954
	P-p38 MAPK of nucleus	PR	0.246	0	2.469	1.524–3.999
Luminal A/B	P-p38 MAPK of cytoplasm	PR	0.292	0.008	2.172	1.225–3.851
	P-p38 MAPK of nucleus	PR	0.308	0	2.952	1.615–5.97
HER2-positive	P-p38 MAPK of cytoplasm	HER2	0.294	0.009	2.145	1.206–3.816
	P-p38 MAPK of nucleus	HER2	0.287	0.03	1.868	1.063–3.281
Triple negative	P-p38 MAPK of cytoplasm/nucleus	None	–	–	–	–

## Discussion

In spit of the important role of p38 MAPK as an important prognostic factor in breast cancer, the expression of P-p38 MAPK in breast cancer and the associated with clinical factors remains to be elucidated due to a lack of sufficient data up to now. In the present study, we found that P-p38 MAPK was expressed in both the cytoplasm and nucleus of breast cancer cells and positively correlated with PR expression in those with the luminal A/B subtype while positively correlated with HER2 expression in those with the HER2-positive subtype only in both correlation analysis and multivariate logistic regression analysis.

We know that MKK3, MKK6 and SEK activate p38 MAP kinase by phosphorylation at Thr180 and Tyr182 together. The P-p38 MAPK mAb we chose has been made by Cell Signaling Technology America Inc. to specifically detect endogenous levels of p38 MAPK only when phosphorylated at Thr180 and Tyr182 (www.cellsignal.com). This antibody was used to detect p38 MAPK (including p38α, β, γ and δ) peculiarly and does not cross-react with the phosphorylated forms of either p42/44 MAPK or SAPK/JNK (Kuroyanagi et al. 2015; Kanno et al. 2005). So our results are based on p38 MAP kinase, including the four subtypes mentioned above, and not a single one of them.

P38 MAPK have been shown to be present in both the nucleus and the cytoplasm of quiescent cells before (Cuenda and Rousseau 2007). Consist with this, our study showed that P-p38 MAPK was expressed not only in the cytoplasm but also in nucleus of breast cancer cells and showed a strongly positive correlation between the expression in cytoplasm and in nucleus (Table 1). Upon cell stimulation, some evidence suggests that p38 MAPK translocates from the cytoplasm to the nucleus (Raingeaud et al. 1995) after phosphorylation while other data are consistent with activation of p38 within the nucleus followed by its movement to the cytoplasm. Generally, the different locations maybe imply their different functions. As such, the exact function of P-p38 MAPK in cytoplasm and nucleus in breast cancer should be gone further study in future.

The aim of our study was to clarify the correlation between P-p38 MAPK expression and clinicopathological parameters through correlation analysis and to further explore possible factors affecting its expression through multivariate logistic regression analysis.

The p38 MAPK signaling pathway plays an important role in breast cancer invasion and metastasis (Han et al. 2007; del Barco and Nebreda 2012). In our study, we found that, none of the various clinicopathological parameters, such as age, menopause status, depth of invasion, histological classification, regional lymph node metastasis, histological type, lymph node metastasis and AJCC stage status, was significantly correlated with P-p38 MAPK expression (Table 2).

Our study also showed that PR was the only one that was positively correlated with P-p38 MAPK in cytoplasm, but not in nucleus, among the three critical factors, ER, PR and HER2 in overall data. As the products of the connection between estrogen and its receptor, PRs only appear in ER+ breast cancer (luminal A/B subtype), as expected, this kind of positive correlation existed only in patients with luminal A/B subtype. PR was also demonstrated the only one which was a significant association in luminal A/B breast cancer in multivariate logistic regression analysis. As members of the steroid hormone receptor family, PRs are able to interact with and activate p38 MAP kinase function as part of signaling cascades (Schiff et al. 2004; Dressing et al. 2009) and might play an important role in breast cancer progression via the MAPK signal pathway (Treviño et al. 2013). This is similar to the fact that steroid hormone-induced rapid activation of MAPK appears to be most robust when both ER and PR are coexpressed (Migliaccio et al. 1998; Ballaré et al. 2003). Endocrine therapy in breast cancer aims to disrupt estrogen stimulation of the cancer cells, which are mediated via binding to the ER/PR. Luminal A/B type breast cancers coexpressing ER and PR are generally more sensitive to endocrine therapy than ER alone and perhaps the “more tumoral sensitivity” maybe partly due to lessening the level of P-p38MAPK through disrupting the hormone receptor PR.

P38 signaling has been verified to drive trastuzumab resistance and invasiveness in HER2-overexpressing breast cancer. At last, we found a positive correlation between P-p38 MAPK expression and HER2 in HER2-positive but not in overall breast cancer, luminal A/B and even in HER2 positive luminal B breast cancer. As contrary, nothing significant correlation was found between P-p38 MAPK expression and PR in HER2 positive luminal B breast cancer. In one hand, these results might advocate that HER2 could play an important role in expression of P-p38 MAPK, and on the other hand, our results might imply that country to HER2, PR might be more important than HER2 in P-p38 MAPK in breast cancer which is worthy of going further study in future.

A lot of publications in the past have shown that development of tamoxifen resistance during the short-time period of neoadjuvant therapy was accomplished by loss of PR and an increase in HER2, as well as activated p38 MAPK (Gutierrez et al. 2005; Linderholm et al. 2011). Probably the up-regulated expression of HER2, which could induce the phosphorylation of p38 MAPK, could also restrain ER/PR expression in luminal B type (Fan et al. 2015; Daniel et al. 2011). These opposite expression of PR and P-p38 that predominately activated by HER2, might be used to interpret not only our finding, but also endocrine therapy resistance in patients with HER2 positive luminal B breast cancer. Even though, both the exact con-relationship between PR and P-p38 MAPK, and the effects of P-p38 MAPK expression on the progress of breast cancer in PR positive luminal A/B breast cancer are still both worthy of going further study in future. In addition, there are many of other markers that may be correlated with the main prognostic, such as CD44+/CD24−/low biomarker (Camerlingo et al. 2014; de Beça et al. 2013), and epidermal growth factor receptor (Cassol et al. 2010), etc. The corelatioship between those markers and P-p38MAPK merits further study in future to explore which one is more effective than the other.

As for the limitation of sample size, larger numbers of patient samples are still required to conclusively determine the biological significance of P-p38 MAPK in patients with invasive breast cancer. Total p38 MAPK also needs to be detected together to show which one is more reliable factor to predict patient survival in future although the active form of p38 MAPK is more really reflect its function than the inactive one in breast cancer.

## Conclusions

P-p38 MAPK was significantly associated with PR in luminal A/B subtype and with HER2 expression in HER2-positive subtype breast cancer. PR/HER2 might association with phosphorylation of p38 MAPK in different subtype breast cancer and further studies will be needed to conform this mechanism. Our study and further mechanism research will not only help us realize the role p38 MAPK play and the infect of PR/HER2 on their phosphorylation, but also could provide more effective means to prevent the progress of tumor in breast cancer.

## Abbreviations

AJCC: American Joint Committee on Cancer
CI: confidence interval
ER: estrogen receptor
HER2: human epidermal growth factor receptor 2
HR: hazard ratio
95 % CI: 95 % confidence interval
OS: overall survival
PR: progesterone receptor
TNM: tumor-node-metastasis
TMA: tissue microarray
P-p38 MAPK: phosphorylated p38 MAP kinase
